# Description of an Extra-Uterine Fœtus, Discharged through the Parietes of the Abdomen

**Published:** 1839-11-30

**Authors:** Robley Dunglison

**Affiliations:** Professor of the Institutes of Medicine, &c. in the Jefferson Medical College


*Description of an Extra-Uterine Fœtus, discharged through the Parietes of the Abdomen.* By Robley Dunglison, M. D., Professor of the Institutes of Medicine, &c. in the Jefferson Medical College.

To the President of the Pathological Society of Phila.

*Girard street*, Nov. 25th, 1839.

Dear Sir,—I was in hopes to be able to attend the meeting of the Pathological Society this evening, and to explain to them the preparation which I sent some time ago, but I find it will not be in my power.

The circumstances of the case were published in the American Medical Intelligencer for May the 15th, of the present year. The woman, from whom the extra-uterine fœtus was taken, was a negress, about 24 or 25 years old. She had previously borne three children, the last about two and a half years ago. Dr. Peyton R. Nelson, of Virginia, was called to her in December last, when she complained of pain in the left side, which had existed for some time. On examination, he found the region considerably enlarged, soft, and compressible, but he was unable to discover the nature of the tumefaction. Six weeks elapsed after this examination, when a tumour formed about an inch to the left of the umbilicus, which, in a day or two, discharged twenty or thirty ounces of a bloody, serous fluid, mixed with purulent matter. Fifteen or twenty days after the first discharge, the head of the fœtal specimen made its appearance, and was extracted gradually in the course of an hour, the aperture through which it had to pass being very small. Dr. Nelson preserved it as well as he was able; still it was considerably injured, as he was obliged to carry it fifteen or twenty miles on horseback.

The specimen, although not unique, is rare. Numerous cases have occurred in which the fœtus, after having undergone a certain degree of development within the uterus, has been discharged, piece-meal, through the parieties of the abdomen, but it has rarely happened that the new being has passed through entire, or nearly so. On this account I have thought that the Society might be desirous to place it amongst the preparations in their Museum.

I am, dear sir,

Respectfully and truly yours,

Robley Dunglison.

